# Association between a Polymorphism of Aminolevulinate Dehydrogenase (ALAD) Gene and Blood Lead Levels in Japanese Subjects

**DOI:** 10.3390/ijerph6030999

**Published:** 2009-03-06

**Authors:** Koichi Miyaki, Htay Lwin, Katsunori Masaki, Yixuan Song, Yoshimitsu Takahashi, Masaaki Muramatsu, Takeo Nakayama

**Affiliations:** 1 Department of Neurology, School of Medicine, Keio University, Tokyo, Japan; 2 Department of Health Informatics, School of Public Health, Graduate School of Medicine, Kyoto University, Kyoto, Japan; E-mails: y-takahashi@umin.ac.jp (Y.T); nakayama@pbh.med.kyoto-u.ac.jp (T.N); 3 Department of Molecular Epidemiology, Medical Research Institute, Tokyo Medical and Dental University, Tokyo, Japan; E-mails: songyixuan1021@yahoo.co.jp (Y.S); muramatsu.epi@mri.tmd.ac.jp (M.M); 4 Department of Internal Medicine, School of Medicine, Keio University, Tokyo, Japan; E-mail: masaki@keiomed.com (K.M); 5 Department of Social and Preventive Epidemiology, School of Public Health, Graduate of School of Medicine, University of Tokyo, Japan; E-mail: hlwinkeiuni08@gmail.com (H.L)

**Keywords:** ALAD gene, blood lead level, environmentally lead exposure, Japanese

## Abstract

This cross-sectional study investigated the relationship between the aminolevulinate dehydrogenase (ALAD) genotype and blood lead levels among 101 Japanese workers. Blood lead concentration measurement, biomarkers, and genotyping were performed. The minor allele frequency (MAF) for ALAD (ALAD2) was 0.08. Although the blood lead level in the subjects with heterozygous GC genotype was significantly higher than those with homozygous GG genotype, there were no significant differences for hemoglobin, hematocrit, serum and urinary ALA levels among genotypes. ALAD2 genotype was significantly associated with the blood lead concentration, even in the environmental lead exposed subjects. Further confirmation with a large sample size is needed.

## 1. Introduction

From the public health point of view, inorganic lead exposure has been a serious problem, not only in the occupational but also in environmental settings [1]. Lead toxication can cause many diseases such as kidney damage [2,3], reproductive system toxicity [4], hypertension [5], and alterations in cognitive development in children, even at low doses [6–8].

Estimation of the health risks associated with low-level exposures to lead has important implications with respect to its regulation. Health-based guidelines limiting occupational and environmental exposures to lead have become more stringent and are now thought to protect most of population against major adverse health effects. Recently the United States Centers for Disease Control and Prevention also recommended the reduction of lead concentrations <10 μg/dL to prevent lead toxicity for children under 6 years old [9]. However, genetically susceptible individuals may not be fully protected by current regulatory standards, so the discovery of genetic factors that might influence susceptibility to lead-induced intoxication could have significant public health ramifications [10].

Polymorphisms of the ALAD gene have been associated with the accumulation of lead in the blood, bone, and internal organs [10], and might differentially predispose for psychiatric symptoms in individuals exposed to lead [11]. Many reports have suggested that the ALAD2 allele may exert protective measures against the neurotoxic effects of lead [12–15]. However, the subjects of these reports have typically been occupationally exposed workers, and no firm evidence exists for an association between ALAD genotype and susceptibility to lead toxicity under environmental exposure levels.

The objective of this study examined the relationship between ALAD1, ALAD2 genotypes and blood lead level among workers who were not occupationally exposed to lead.

## 2. Methodology

### 2.1. Subjects

We explained this study to Japanese healthy workers from a Japanese chemical industry company located in Kanagawa prefecture, and received a written informed consent from each participant. A total of 101 healthy workers (73 men and 28 women, after exclusion of those who occupationally handled or used to handle lead in their lives) were subjected to medical examination and genotyping. Among these, 41 men were engaged in logistics and the rest of them were engaged in office work. The mean age, and body mass index (BMI) were 43.4±11.9 years and 23.4±4.13 kg/m^2^ (mean ± SD), respectively.

### 2.2. Basic Characteristics and Blood Measurements

Age and smoking status were self-reported. The medical and occupational histories were established by interview. Height, weight, systolic and diastolic blood pressures were measured by trained staff nurses. BMI was calculated as weight by height square (kg/m^2^).

The blood samples of all participants were taken by trained staff nurses and the withdrawn blood samples were kept in the plain tubes for examining fasting blood glucose, serum lipids (total cholesterol, triglyceride, HDL cholesterol), aspartate aminotransferase (AST), alanine aminotransferase (ALT), gamma-glutamyl transpeptidase (γ-GTP), blood urea nitrogen (BUN), serum creatinine (Cr), serum uric acid (UA), blood lead concentration, and biomarkers for lead exposure (serum and urinary 5-aminolevulinic acid; sALA and uALA, and Zinc protoporphyrin; ZnPP).

Blood lead concentration was measured by flameless atomic absorption spectrophotometry (Hitachi Z-9000) with Zeeman effect background correction. To determine delta-aminolevulinic acid levels (sALA in serum and uALA in urine) and Zinc protoporphyrin (Znpp) in blood, we used a type L-6200 HPLC (Hitachi, Japan). Using this apparatus, stopped-flow HPLC was applied to determine these parameters using a reverse phase column after a simple pretreatment. The highest fluorescent intensity was obtained when the pretreated sample was doubly diluted with 100 mM sodium acetate (pH 5.0), and the sample (60 μL) was introduced into the condensing coil at a temperature of 98 °C, then 50% acetylacetone in 25% ethanol was added, followed by secondary mixing with 10% formaldehyde solution. The detection limit was 2 μg/L, which was 2.5 times higher than that achievable by conventional method. Relative standard deviations of 10 blood samples calculated from 4 determinations per sample for 1 week were within 5%. Haemoglobin was determined by the cyanomethaemoglobin method.

### 2.3. Genotyping

The peripheral blood from all participants were drawn, and genotyping for the ALAD polymorphism (a single nucleotide polymorphism database, dbSNP ID: rs1800435 referenced information from NCBI, and the International HapMap Project) was performed by polymerase chain reaction (PCR) and the single nucleotide primer extension (SNuPe) methods. The blood was pretreated with Ampdirect (SHIMADZU Corporation, Kyoto, Japan), which could eliminate the DNA extraction process and amplify the genomic DNA directly from the whole blood. PCR primers 5’ - GGCCTCAGTCTTCCCTCCTA -3’ (sense) and 5’ -ACCTCTCCACCTCCCGAGTA -3’ (antisense) as well as two versions of SNuPe primers (5’ - CCACACAGGTATGGTGTGAA -3’ and 5’ -CCACACAGGTACGGTGTGAA -3’) were designed with DNASIS Pro Ver.2.0 (Hitachi Software Engineering Co. Ltd., Tokyo, Japan), since there is another SNP site 9-bp upstream of the ALAD1-2 polymorphism. Then, mixed SNuPe primer (5’ -CCACACAGGTAYGGTGTGAA -3’) was made. The PCR reactions began with preheating at 80 °C for 15 minutes, followed by denaturing at 94 °C for 4.5 minutes and 40 cycles of denaturing at 94 °C for 30 seconds, annealing at 60 °C for 1 minute, extension at 72 °C for 1 minute, with a final extension at 72 °C for 7 minutes. We then carried out the SNuPe method. The reactions included 25 cycles of denaturing at 94 °C for 10 seconds, annealing at 53 °C for 5 seconds, and extension at 60 °C for 10 seconds. We analyzed the products by using ABI 7700 (Amersham Biosciences Corp.). As for genotyping accuracy, we compared the genotyping results by SNuPe method as well as conventional RFLP, and the agreement rate was 100% except one, which was not determined due to the lack of residual sample.

### 2.4. Statistical Analysis

All values were shown by mean and Standard deviation (SD) except the value mentioned with the Geometric mean (GSD). We compared the mean value of all basic characteristic, clinical, biochemical variables including blood lead level between GG and GC genotypes by Student's t-test or χ^2^ test. The mean value of biomarkers for lead exposure between two genotypes of ALAD gene was compared by using Student’s t-test. All *P* value ≤0.05 was considered as statistical significant.

## 3. Results and Discussion

**Figure 1.**
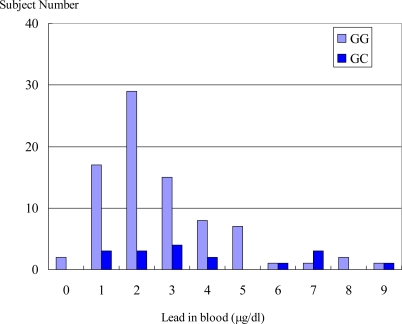
Histogram of blood lead in all non-occupationally lead exposed participants. Vertical axis showed number of subjects divided by 10 individual each. Longitudinal axis showed concentration of blood lead (μg/dl). Vertical bar (light blue) showed GG genotype of ALAD gene, and vertical bar (dark blue) showed GC genotype of ALAD gene.

Table 1 shows the basic characteristics of occupationally non lead exposed subjects. The mean (SD) age of all study participants is 43.4 (11.9) yr. The mean blood lead level of all subjects was 3.38 μg/dL and the mean blood lead level of GC genotype was significantly higher than that of GG homozygous genotype. Many previous studies have shown that the ALAD genotype was associated with blood lead levels among environmentally exposed children and occupationally exposed lead workers [16,17]. Subjects with ALAD heterozygous GC, and homozygous CC genotypes were more susceptible to increased blood lead levels than those with the homozygous GG genotype. ALAD2 carriers had significantly higher blood lead level that ALAD1 homozygous [16–19]. The hypothesis of mechanism for lead binding affinity with ALAD2 remained controversial [15,20]. However, some studies found no significant association between ALAD2 carriers and blood lead level [21–23]. Although the association result is inconsistent, our study found the blood lead level in the subjects with heterozygous GC genotype was significantly higher than that in those with homozygous GG genotype, even in the environmentally same exposed lead condition (i.e. very low lead exposure naturally). Age and smoking status were not significantly different between GG and GC genotypes in the present study (Table 1), where these were known confounding variables for ALAD enzyme activity [24,25]. Furthermore, in Figure 1, the prevalence of blood lead levels from 1μg/dL to 5 μg/dL in GG genotype was much higher than that in GC genotype, but a 7μg/dL blood lead level was more prevalent in GC than that in GG genotype. Even in the all non-occupationally lead exposed participants, the blood lead level was shifted from the left to the right in ALAD2 carrier genotype (GC). Our finding is similar to that of the other studies [16–19].

Our study showed that the mean white blood cell count in GC genotype was significantly higher than that in GG genotype (Table 1), but Shaik *et al.* [23] found no significant association. Exploration of this discrepancy might be interesting in a future longitudinal study, although the mechanism of this relationship could not be explained in our recent cross-sectional study.

The recent study found that the frequency of GG genotype of ALAD was 83.2 %, and that of GC genotype of ALAD was 16.8 % in this non-occupationally lead exposed Japanese population. There was no CC homozygous genotype in this Japanese general population. The frequency distribution of genotype was in Hardy–Weinberg equilibrium. The International HapMap Project, and Kelada *et al.* [15] also reported that ALAD2 allele frequency is high in Caucasian, but low in Asians, including Chinese and Japanese, and African populations, and a few or no ALAD2 homozygotes was found in such populations. Genotype frequency distribution in the present study was similar with data from the international HapMap for Japanese. According to this data set, the major allele frequency of ALAD (ALAD1) was 0.92 and the minor allele frequency (MAF) for ALAD (ALAD2) was 0.08. Although our study population is the non-occupationally lead exposed subjects, the frequency of MAF was very similar to that of the general Japanese population according to the International HapMap Project data, as well as the study of Sakai *et al.* [26] that reported on an high occupational lead exposed subject. Thus our study not only confirmed the allele and genotype frequencies of ALAD2 even in the non-occupationally lead exposed free living Japanese population, but also the ALAD2 allele was rare in Japanese population. It seems to be concluded that the ALAD2 allele is rare in Asian populations including a Taiwanese population [15,21]. This finding may at least partly provide support for a explanation about that why the blood lead levels in the general population with environmental exposure to lead level are lower in Japan and other Asian countries when compared with the European countries [27].

Table 2 shows the concentration of biomarkers for lead exposure, in non-occupationally lead exposed subjects. There were no significant differences for serum and urinary ALA, and ZnPP between the subjects with GG and GC genotype. The hematopoietic system is one of the target organs in lead poisoning. One of the most important mechanisms of lead toxicity is its effect on enzymes in the heme biosynthetic pathway. The enzyme in the biosynthetic pathway of heme in which the effects of lead are of the highest clinical interest is ALAD. When ALAD activity is deficient due to lead poisoning, erythrocyte synthesis become inhibited and blood hemoglobin concentrations become lower. In the second step of heme synthesis, ALAD catalyzes the formation of porphobilinogen from two molecules of ALA. ALAD is the most sensitive enzyme to lead in the heme pathway and has a high affinity for the metal. Lead binds the enzyme’s SH group, which normally binds zinc, preventing the binding of ALA [28]. Because lead effectively inhibits ALAD activity, resulting in accumulation of ALA in blood and urine, urinary ALA has also been used as a biomarker for lead exposure or a marker of early biologic effect of lead [28,29]. In this study, we did not find any significant differences for blood hemoglobin, hematocrit, red blood cell count, ZnPP, and serum and urinary ALA levels between GG, and GC genotype of ALAD. These may be explained by the fact that our study subjects were all adults, and who were only exposed to lead environmentally unlike chronically exposed lead workers, and the mean value of blood lead level in this study subject with ALAD carrier (GC genotype) was only 4.5 μg/dL, which was lower than the minimum level for lead poisoning (<10 μg/dL for children under 6, recommended by CDC [9]). So, these findings might be resulting from the low blood lead levels, which may not induce hemopoietic abnormalities and consequently not inhibit ALAD activity, effectively.

## 4. Conclusions

This is the first descriptive report of the association between ALAD2 genotype and blood lead levels among non-occupationally lead exposed Japanese subjects, although there were some limitations such as lack of data for alcohol consumption and ALAD activity level. The present findings as well as lead toxicity complications will be examined in further longitudinal studies.

## Figures and Tables

**Table 1.** Basic characteristics of non-occupationally lead exposed subjects.

	All non lead exposed participants (N=101)	ALAD genotype		*P*
		GG (N=84)	GC (N=17)	
*Demographics and Clinical*				
Sex (Male/Female)	73/28	61/23	12/5	0.54
Age (years)	43.4 (11.9)	43.2 (11.9)	44.5 (12.2)	0.68
Smoking (Yes/No)	55/46	45/39	10/7	0.81
Height, cm	166.3 (8.3)	166.4 (8.2)	165.5 (9.2)	0.66
Weight, kg	65.0 (13.7)	65.3 (14.4)	63.8 (9.6)	0.68
Body mass index, kg/m^2^	23.4 (4.1)	23.4 (4.2)	23.3 (3.6)	0.94
SBP, mmHg	130.2 (18.8)	128.7 (17.4)	137.7 (23.9)	0.15
DBP, mmHg	78.8 (13.0)	78.3 (12.6)	81.1 (15.1)	0.43
*Complete Blood Counts*				
White blood cell count, 10^3^/μL	61.2 (14.7)	59.7 (13.8)	68.4 (17.6)	0.02
Red blood cell count, 10^6^/μL	465.6 (41.6)	465.1 (42.2)	468.1 (40.0)	0.79
Hemoglobin, g/dL	14.4 (1.6)	14.3 (1.6)	14.7 (1.7)	0.37
Hematocrit, %	43.7 (4.0)	43.6 (4.0)	44.1 (4.3)	0.65
Platelet count, 10^3^/μL	24.9 (4.9)	24.9 (4.6)	25.0 (6.1)	0.94
*Biochemical*				
AST/GOT, IU/l ^#^	22.2 (1.4)	22.1 (1.4)	22.4 (1.4)	0.90
ALT/GPT, IU/l ^#^	22.7 (1.8)	22.8 (1.9)	22.0 (1.6)	0.81
γ-GTP, IU/l	34.0 (2.1)	32.8 (2.0	40.9 (2.6)	0.38
Total cholesterol, mg/dL	199.6 (36.3)	196.9 (35.1)	212.8 (40.1)	0.10
Triglyceride, mg/dL ^#^	105.9 (1.8)	105.7 (1.9)	107.1 (1.8)	0.93
HDL cholesterol, mg/dL ^#^	53.4 (1.3)	52.9 (1.3)	55.6 (1.3)	0.45
Blood urea nitrogen, mg/dL	13.6 (3.5)	13.6 (3.6)	13.5 (2.8)	0.90
Serum creatinine, mg/dL	0.81 (1.2)	0.81 (1.2)	0.78 (1.2)	0.40
Serum uric acid, mg/dL	5.34 (1.3)	5.38 (1.3)	5.12 (1.2)	0.45
Lactate dehydrogenase, IU/l ^#^	181.6 (1.1)	180.9 (1.2)	185.2 (1.1)	0.54
Fasting blood sugar, mg/dL ^#^	95.6 (1.2)	96.2 (1.2)	92.8 (1.2)	0.49
Fasting blood sugar, mg/dL ^#^	4.98 (1.1)	4.96 (1.1)	5.09 (1.2)	0.44
*Outcome index*				
Blood lead level, μg/dL	3.38 (1.9)	3.18 (1.8)	4.35 (2.4)	0.02

All values are mean (SD) except the value mentioned with the specific symbol; # Geometric mean (GSD)

BMI is calculated as weight (kg)/height (m^2^)

SBP: Systolic blood pressure, DBP: Diastolic blood pressure

AST/GOT:aspartate aminotransferase/glutamic oxaloacetic transaminase.

ALT/GPT: for alanine aminotransferase/glutamic pyruvic transaminase.

γ-GTP: gamma-glutamyl transpeptidase

HDL cholesterol: High density lipoprotein cholesterol

*Significant P value <0.05 in Student's t-test or χ^2^ test.

**Table 2.** Biomarkers of lead exposure among non-occupationally lead exposed Japanese.

Variable	ALAD1 (GG)	ALAD2 (GC)	*P*
Blood lead level, μg/dL	3.2 ± 0.19	4.4 ± 0.58	0.02
sALA, ng/mL	9.1 ± 0.39	10.2 ±0.91	0.28
uALA, mg/g creatinine	0.85 ± 0.05	0.81 ± 0.11	0.70
ZnPP, μM	19.0 ± 0.54	21.2 ± 1.0	0.05
Hb, mg/dL	14.3 ± 1.6	14.7 ± 1.7	0.37

All value are shown as mean ± SD

sALA: Serum 5-aminolevulinic acid,

uALA: Urinary 5-aminolevulinic acid

ZnPP: Zinc protoporphyrin, Hb: Hemoglobin
